# Amelanotic Uveal Melanomas Evaluated by Indirect Ophthalmoscopy Reveal Better Long-Term Prognosis Than Pigmented Primary Tumours—A Single Centre Experience

**DOI:** 10.3390/cancers14112753

**Published:** 2022-06-01

**Authors:** Anna Markiewicz, Piotr Donizy, Monika Nowak, Mateusz Krzyziński, Martyna Elas, Przemysław M. Płonka, Jolanta Orłowska-Heitzmann, Przemysław Biecek, Mai P. Hoang, Bożena Romanowska-Dixon

**Affiliations:** 1 Department of Ophthalmology and Ocular Oncology, Faculty of Medicine, Jagiellonian University Medical College, 31-501 Krakow, Poland; b.romanowska-dixon@uj.edu.pl; 2 Department of Ophthalmology and Ocular Oncology, University Hospital, 31-501 Krakow, Poland; monowak@su.krakow.pl; 3 Department of Clinical and Experimental Pathology, Division of Clinical Pathology, Wroclaw Medical University, 50-556 Wroclaw, Poland; piotrdonizy@wp.pl; 4 Faculty of Mathematics and Information Science, Warsaw University of Technology, 00-628 Warsaw, Poland; mateusz.krzyzinski.stud@pw.edu.pl (M.K.); przemyslaw.biecek@mini.pw.edu.pl (P.B.); 5 Department of Biophysics and Cancer Biology, Faculty of Biochemistry, Biophysics and Biotechnology, Jagiellonian University, 30-387 Krakow, Poland; martyna.elas@uj.edu.pl (M.E.); przemyslaw.plonka@uj.edu.pl (P.M.P.); 6 Department of Pathomorphology, University Hospital in Krakow, 30-688 Krakow, Poland; jolanta_heitzman@op.pl; 7 Department of Pathology, Massachusetts General Hospital, Harvard Medical School, Boston, MA 02114, USA; mhoang@mgh.harvard.edu

**Keywords:** pigmentation, uveal melanoma, amelanotic, ocular melanoma, melanoma, melanoma pigmentation, melanin, prognosis, amelanotic melanoma

## Abstract

**Simple Summary:**

The prognosis of uveal melanoma is dependent on many factors. Among the most relevant are the tumour size, ciliary body involvement, extraocular extension, tumour cell type, genetic abnormalities, and the presence of lymphocytic infiltration. The assessment of some of these is very expensive and not available in every centre. In the search for another prognostic factor, we decided to focus on the degree of tumour pigmentation, which is very easy to assess on indirect ophthalmoscopy. It was demonstrated that patients with amelanotic uveal melanomas (those without pigment) lived longer and the eventual spread of the neoplastic process occurred later than in patients with heavily pigmented tumours. In heavily pigmented uveal melanomas, we found features on histopathological examination that were associated with an unfavourable prognosis. In the two separate groups of uveal melanomas with different degrees of pigmentation, we observed that amelanotic tumours with a lower clinical stage had the best prognosis.

**Abstract:**

(1) Background: There is a constant search for new prognostic factors that would allow us to accurately determine the prognosis, select the type of treatment, and monitor the patient diagnosed with uveal melanoma in a minimally invasive and easily accessible way. Therefore, we decided to evaluate the prognostic role of its pigmentation in a clinical assessment. (2) Methods: The pigmentation of 154 uveal melanomas was assessed by indirect ophthalmoscopy. Two groups of tumours were identified: amelanotic and pigmented. The statistical relationships between these two groups and clinical, pathological parameters and the long-term survival rate were analyzed. (3) Results: There were 16.9% amelanotic tumours among all and they occurred in younger patients (*p* = 0.022). In pigmented melanomas, unfavourable prognostic features such as: epithelioid cells (*p* = 0.0013), extrascleral extension (*p* = 0.027), macronucleoli (*p* = 0.0065), and the absence of BAP1 expression (*p* = 0.029) were statistically more frequently observed. Kaplan–Meier analysis demonstrated significantly better overall (*p* = 0.017) and disease-free (*p* < 0.001) survival rates for patients with amelanotic tumours. However, this relationship was statistically significant for lower stage tumours (AJCC stage II), and was not present in larger and more advanced stages (AJCC stage III). (4) Conclusions: The results obtained suggested that the presence of pigmentation in uveal melanoma by indirect ophthalmoscopy was associated with a worse prognosis, compared to amelanotic lesions. These findings could be useful in the choice of therapeutic and follow-up options in the future.

## 1. Introduction

Uveal melanoma is the most common primary intraocular neoplasm in adults (2–11 new cases per 1,000,000/1 year) and is sometimes diagnosed in children [1,2,3,4,5]. Its incidence depends on many factors. Caucasian race, age over 60, residence at higher latitudes, dysplastic nevus syndrome, presence of choroidal nevi, light irises, darker choroidal pigmentation, or ocular melanocytosis predispose to this type of malignancy [5,6,7,8,9]. Conservative treatment is most commonly used in the treatment of uveal melanoma. However, in some cases, surgical treatment in the form of endoresection or exoresection is preferable. Further, in cases of very large tumours, tumour involvement of the optic disc or ring melanoma, enucleation is usually performed [5,10]. The conservative treatments most commonly used for uveal melanoma include brachytherapy with isotopes 125I, 103Pd, 131Cs, 106Ru, and 90Sr. Less commonly, patients are treated with proton beam radiotherapy. Both methods manifest similar results of local control, at the level of 95–98% [11,12,13,14,15,16,17].

Unfortunately, it is estimated that in 50% of patients, despite positive local treatment results, the neoplastic process spreads. Most metastases are located in the liver (>90%), followed by the lungs, bones, subcutaneous tissue, etc. [5,6,18]. Despite numerous advanced studies, there is still no effective treatment for generalized dissemination. The ongoing clinical trials of new therapies show only prolonged survival, not recovery [19,20].

The prognosis of uveal melanoma depends on many factors. Older age (>60 years), large tumour size, ciliary body involvement, extraocular extension, epithelioid cell type, genetic abnormalities (chromosome 3 monosomy, gain 8q), and lymphocytic infiltration worsen the prognosis [5,6].

Tumour pigmentation is also considered by some a poor prognostic factor [6,21,22,23,24,25,26]. Reports describing tumour pigmentation and its impact on survival are mainly based on histopathologic evaluation of uveal melanoma specimens. The Collaborative Ocular Melanoma Study Group (COMS) classification of the pigmentation of medium and large tumours is shown in Table 1. Small tumours were not investigated in the COMS study since eyeballs with small lesions were not removed [21,27]. In turn, Markiewicz et al. evaluated the percentage content of melanin in all the histopathological specimens, distinguishing three groups depending on the melanin level: 0–10 (predominantly-amelanotic), 11–50 (medium-pigmented), and 51–100 (with strong pigmentation) [28].

According to the COMS study, large tumour size, epithelioid cell type, and higher incidence of necrosis (66.2%) were associated with heavy pigmentation [27].

McLean et al., Seddon et al., and Shammas and Blodi demonstrated a trend toward increased mortality in heavily pigmented tumours in their study. That said, in the McLean et al. study, the increase in pigmentation was not a significant factor in large epithelioid cell tumours [21,22,23,24].

Clinically, the tumours range from light yellow, referred to as amelanotic, to dark brown. Shields et al. analyzed a group of 8100 cases of uveal melanoma and distinguished three grades of pigmentation in ophthalmoscopy: pigmented, nonpigmented, and mixed, and showed that the ability to metastasize was greater in tumours showing the presence of pigment regardless of race [18].

While the diagnosis of a pigmented uveal melanoma based on the eye fundus examination is not problematic in everyday practice, an amelanotic tumour, especially a small one, can be quite challenging. Nonpigmented uveal melanomas should be differentiated from other intraocular lesions, both malignant and benign such as: amelanotic nevus, metastasis, hemangioma, peripheral exudative hemorrhagic chorioretinopathy, sclerochoroidal calcification, osteoma, granuloma, lymphoma, solitary idiopathic choroiditis [29,30]. Fortunately, today, in doubtful cases, ultrasound and often optical coherence tomography (OCT) provide information for the correct diagnosis. According to COMS, the accuracy of diagnosis based on clinical examination is over 99% [31]. Amelanotic melanomas account for 20–25% of all melanomas and are thought to arise from an amelanotic nevus [21,32].

Both cutaneous melanoma and uveal melanoma derive from melanocytes arising from melanoblasts developing from neural crest. Despite this, the two cancers show major differences in genetic background, mutational burden, clinical presentation, and response to systemic treatment [33].

Among studies that address the effect of cutaneous melanoma pigmentation on the prognosis, there are those that show a higher mortality rate when amelanotic lesions are present [34].

Due to the still not fully explored role of pigmentation and the presence of studies suggesting the prognosis and response of tumours to treatment may depend on their degree of pigmentation, we decided to analyze whether the degree of uveal melanoma coloration in clinical assessment affects the prognosis of this neoplasm. To assess the degree of uveal melanoma pigmentation, we chose the simplest and most commonly used test: indirect ophthalmoscopy. We wanted to check whether the assessment of uveal melanoma pigmentation should become an important element of the ophthalmic examination in ocular oncology.

## 2. Materials and Methods

The study group consisted of 154 patients diagnosed in 2002–2011 with uveal melanoma treated by primary enucleation at the Department of Ophthalmology and Ocular Oncology, University Hospital. A prerequisite for inclusion in the analyzed group was the presence of clinical evaluation of uveal melanoma pigmentation before treatment was undertaken. Patients were enrolled in the study based on the availability of their medical records and tissue specimens. Clinical data were retrieved from the archived medical records, and details of diagnostic and therapeutic procedures performed were sourced from the Ocular Oncology Outpatient Clinic.

Records were reviewed for clinical and pathological data including age, sex, affected eye, largest basal diameter and thickness of the tumour, tumour staging (primary tumour (pT) and American Joint Committee on Cancer (AJCC) prognostic stage), tumour location relative to the equator, ciliary body involvement, tumour pigmentation and shape, concomitant glaucoma/retinal detachment, haemorrhage, histological subtype, scleral/optic nerve infiltration, as well as tumour necrosis. Additionally, detailed histological parameters, such as mitotic rate, nucleoli size, presence of tumour-infiltrating lymphocytes (TILs, evaluated semi-quantitatively based on HE slides with further division into two subgroups: no TILs or presence of any TILs), scleral and tumour angioinvasion by melanoma cells, as well as BAP1 (encoding BRCA1-associated protein 1; located on chromosome 3) status evaluated by immunohistochemistry were considered. The largest basal diameter, height of the tumour and stage were described in line with the AJCC guidelines [35,36]. Demographic, clinical and histopathological data of the analyzed group of patients are presented in Table 2 and Table 3.

### 2.1. Clinical Evaluation

Clinical examination included eye slit lamp biomicroscopy and ophthalmoscopy. Tumour dimensions were assessed with ultrasonography. The tumour thickness (from the internal scleral surface to its apex) and the biggest basis diameter were measured (Quantel Medical, ABSolu, France). Screening for metastatic disease in all cases involved serum biochemistry, liver function tests, ultrasonography or computer tomography, or magnetic resonance of the liver and chest X-ray picture.

### 2.2. Evoluation of Tumours Pigmentation

**Figure 1:**
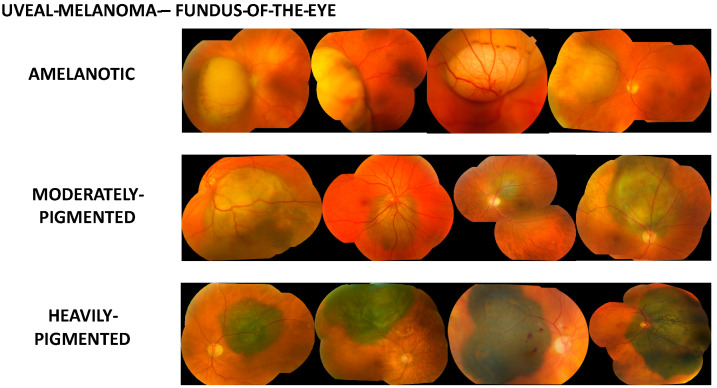
Clinical examples of the different pigmentation of uveal melanomas in each of categories assessed in the indirect ophthalmoscopy.

Data on the assessment of uveal melanoma pigmentation on fundus examination have been recorded in our clinic for several decades. Initially, this was in the form of a description, and then for more than 20 years, in special forms filled out during visits. Evaluation of tumour pigmentation was first performed in the outpatient clinic and then in the clinical department during the ophthalmological examination before the planned surgery (in this case, removal of the eyeball). The examination was carried out by specialists in ophthalmic oncology using indirect ophthalmoscopy. Degree of pigmentation in each tumour was graded quantitatively by sorting into three groups that represented amelanotic, moderate, and heavy pigmentation. The amelanotic was white-yellow and no pigment was found in its area. The heavily pigmented tumour took on a dark grey, graphite, brown, or even black colour. Alternatively, the moderately pigmented are all tumour colorations located between amelanotic and heavy pigmented. Figure 1 shows examples of the pigmentation of uveal melanomas in each of these categories.

In our analysis, clinical examination including assessment of tumour pigmentation was carried out by at least by two independent ophthalmologists, specialising in ocular oncology.

Based on our own long-term experience, which indicated that in clinical assessment there may be difficulties in categorising tumours in terms of pigmentation, we decided to carry out the analysis only by dividing them into two main groups. One group consisted of lesions lacking pigmentation—amelanotic, and the other of those that showed any pigmentation. In our daily practice, the biggest problems (especially for inexperienced doctors) were very poorly pigmented lesions, which were incorrectly classified as amelanotic, as well as tumours with quite strong pigmentation, which should be classified as moderately pigmented, but were classified as heavy pigmented. In our opinion, physicians who did not deal with ocular oncology on a daily basis would find it easier to assess the prognosis of uveal melanoma according to pigmentation only when dividing them into the two categories described above. The percentage of melanin on histopathological examination was assessed as described in the introduction in [28].

### 2.3. Immunohistochemistry

Immunohistochemical analysis was performed on four µm-thick paraffin sections mounted on silanized slides (Agilent DAKO, Santa Clara, CA, USA) using standard immunohistochemistry techniques, heat-induced epitope retrieval with EnVision Target Retrieval Solution (Agilent DAKO, Santa Clara, CA, USA), and primary antibody against BAP1 (sc-28383 (C-4), dilution 1:100, Santa Cruz Biotechnology, Dallas, TX, USA).

### 2.4. Statistical Analysis

Statistical analysis was performed using the R language and the survminer tool (available online: https://www.rproject.org/ (accessed on 15 January 2022)) [37,38]. Overall survival (OS) was defined as the time period from the date of UM diagnosis until death date or the last follow-up and disease-free survival (DFS) as the time from finishing UM treatment until metastasis or the last follow-up. In order to determine the OS and DFS rates, Kaplan–Meier curves and the log-rank test were used; all analyses were carried out using the survival package for R. In order to determine the correlations between the degree of pigmentation and continuous variables, the Wilcoxon two-sample test was used. The correlations between the presence of the degree of pigmentation and categorical variables were determined using Fisher’s exact test. A *p*-value below 0.05 was considered significant for all comparisons.

## 3. Results

Based on indirect ophthalmoscopy, in the group of patients analyzed there were 26 (16.9%) amelanotic and 128 (87.1%) pigmented uveal melanomas, of which 61 (39.6%) were moderately pigmented and 67 (43.5%) heavily pigmented.

### 3.1. Clinical Pigmentation of Uveal Melanoma—Correlations with Clinical Parameters

Amelanotic tumours were significantly more common in the younger patient group (*p* = 0.022) (Table 2).

Mushroom shape tumour with Bruch’s membrane rupture in ophthalmoscopy and ultrasound imaging was found more frequently in amelanotic than in pigmented uveal melanomas (*p* = 0.033) (Table 2).

### 3.2. Clinical Pigmentation of Uveal Melanoma—Correlations with Histopathological Parameters

In cell type analysis, no case of amelanotic uveal melanoma showed epithelioid cell type. On the contrary, epithelioid cells were significantly more frequently found in pigmented melanomas (mixed type 78 (60.94%) and epithelioid 26 (20.31%)) (*p* = 0.001). 

We found a significant correlation between clinical and histopathologic assessment (*p* < 0.001). A total of 57/58 (98.3%) of heavily pigmented tumours assessed by microscopic evaluation were described as pigmented tumours by indirect ophthalmoscopy. A number of 8/13 (61.5%) of microscopically amelanotic tumours were also evaluated as tumour with no melanin in clinical examination.

The presence of extrascleral extension was observed more frequently in pigmented uveal melanoma (*p* = 0.027).

In pigmented uveal melanoma, prominent nucleoli (macronucleoli) were significantly more frequent than in amelanotic (*p* = 0.0065).

Analysis of BAP1 in both tumour groups showed that the absence of immunohistochemical BAP1 expression was significantly more frequent in pigmented uveal melanoma (*p* = 0.029) (Table 3).

### 3.3. The Effect of Clinical Pigmentation of Uveal Melanoma on Long-Term Survival—Kaplan-Meier Analysis

**Figure 2:**
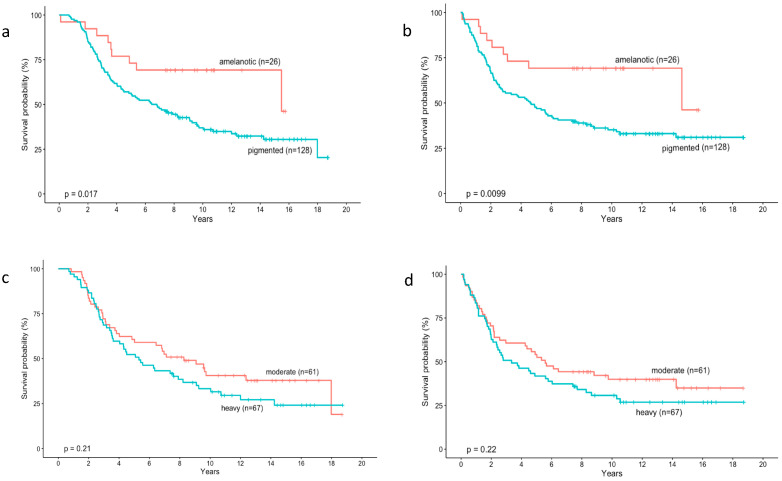
Kaplan–Meier analysis of the prognostic impact of pigmentation degree assessed with indirect ophthalmoscopy in patients with uveal melanoma. Overall survival (**a**) and disease free survival (**b**) were significantly longer in patients with amelanotic tumours. No significant differences in overall survival (**c**) and disease free survival (**d**) between moderately and heavily pigmented tumours were observed.

In the analyzed group of patients, overall survival (*p* = 0.017) and disease free survival (*p* = 0.0099) were significantly longer in patients with amelanotic tumours (Figure 2a,b).

Interestingly, we did not observe significant differences in overall survival (*p* = 0.21) and disease free survival (*p* = 0.22) between moderately and heavily pigmented tumours (Figure 2c,d).

**Figure 3:**
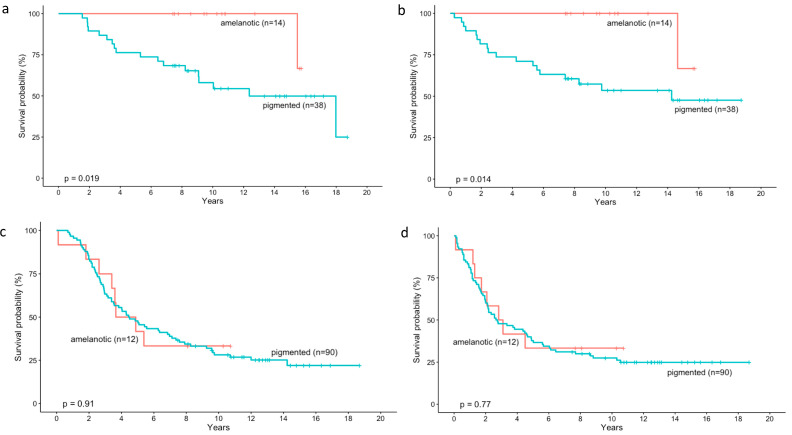
Kaplan–Meier analysis of the prognostic impact of pigmentation degree assessed with indirect ophthalmoscopy in patients with uveal melanoma according to the AJCC classification. Pigmentation affects overall survival (**a**) and disease free survival (**b**) in lower stage (II, both IIA and IIB). Patients with amelanotic tumours live significantly longer, as well as have the lower risk of metastases, than patients with pigmented tumours. In contrast, the degree of tumour pigmentation has no effect on overall survival (**c**) and disease free survival (**d**) in higher disease stages (III, both IIIA and IIIB and IIIC).

When analyzing whether pigmentation affects overall survival (*p* = 0.019) and disease free survival (*p* = 0.014) according to clinical stage according to the AJCC, we found that in lower stage (II, both IIA and IIB) patients with amelanotic tumours live significantly longer, as well as have a lower risk of metastases, than patients with pigmented tumours (Figure 3a,b).

In contrast, the degree of tumour colouration has no effect on overall survival (*p* = 0.91) and disease free survival (*p* = 0.77) in higher disease stages (III, both IIIA and IIIB and IIIC) according to the AJCC classification (Figure 3c,d).

## 4. Discussion

Amelanotic uveal melanomas accounted for 16.9% of all tumours we analysed whose pigmentation was assessed by eye fundus examination with an indirect ophthalmoscopy. This result was similar to the reports of other authors, in whose studies amelanotic melanomas occurred in approximately 20% [21,32].

We noted that amelanotic tumours were significantly more common in the younger patient group (*p* = 0.022). This may suggest that they may have a better prognosis as is the case in children and young adults [4]. The lower risk of metastases at a younger age of onset was confirmed by Shields et al. based on multivariate analysis (*p* < 0.001) [18].

Studies that analysed the effect of uveal melanoma pigmentation on prognosis were mainly based on analyses of histopathological specimens. Studies by McLean et al., Seddon et al., Shammas and Blodi, and COMS have shown a trend toward increased mortality in heavily pigmented tumours. Furthermore, Shammas and Blodi estimated the mortality rate for amelanotic melanomas to be 19%, for intermediate pigmented 39%, and 65% for heavily pigmented.

According to COMS, large tumour size, epithelioid cell type and higher incidence of necrosis (66.2%) were also associated with heavy pigmentation [27].

In our analysis, we noted similar correlations. The presence of epithelioid cells was significantly more frequent in pigmented uveal melanoma (*p* = 0.001). Furthermore, what is most interesting is that in no case of amelanotic melanoma was the epithelioid cell type present, regardless of size. The same observation was noted by the authors of the COMS report, who found no single case of epithelioid cell melanoma even among large tumours [27]. Similar to the COMS report, in our group necrosis tended to be more frequent in pigmented than in amelanotic tumours (11.72% vs. 3.85%, respectively), but without statistical significance.

Analyzing other histopathological features, we found that features that indicated greater malignancy of the tumour were significantly more common in pigmented uveal melanoma. These features were extrascleral growth (*p* = 0.027) and prominent nucleoli (macronucleoli) (*p* = 0.0065).

It is well known that a somatic mutation causing inactivation of BAP1 (encoding BRCA1-associated protein 1; located on chromosome 3) in uveal melanoma cells was prognostically unfavourable [5,6]. In our study, the analysis of BAP1 in both tumour groups showed that the absence of BAP1 expression was significantly more frequent in pigmented uveal melanoma (*p* = 0.029).

All of the above observations would suggest that pigmented uveal melanomas should have a worse prognosis. This remains in apparent opposition to the common opinion regarding melanoma that amelanotic tumours have a poorer prognosis than pigmented tumours [36].

The reason for such contrasting observations may be the ambivalent role of melanin and melanogenesis in the development of melanoma [39].

Melanogenesis is a symptom of cellular differentiation, a process opposite to cancerogenesis and from this point of view, melanogenesis should indicate a good prognosis for melanoma patients [38]. However, numerous recent review articles gather examples suggesting that melanogenesis actually indicates progression of the tumour [40,41]. It mainly concerns patients with skin melanoma; the present study on uveal melanoma adds new data on this problem.

A putative function of melanin—protection against free-radical damage [42] as well as the ability to bind toxins, antibiotics, and other chemicals denominated commonly as anticancer drugs (chemotherapeutica) [43,44] determine the extraordinary resistance of melanomas to therapy [45,46,47,48].

Melanin modifies the answer of melanocytes to biological factors (e.g., active factors of the immunological system determining cellular apoptosis or proliferation) [49,50].

Melanin and melanogenesis itself may produce toxic factors (free radicals) [51], and activation of biochemical pathways activating melanogenesis may at the same time activate pathways leading to increased proliferation or inhibition of apoptosis [52,53]. Finally, melanin remarkably modifies the mechanical properties of melanoma cells, thus affecting its invasiveness and metastasizing [54,55].

This process can be facilitated during tumour progression in positive feedback [39]. 

Another reason for the presence of melanin that correlates with the worse prognosis may be that melanogenesis is related to significant upregulation of hypoxia-dependent pathways, which contribute to the production of a more aggressive phenotype of melanoma. Furthermore, a higher melanin content and a disproportion of eumelanin to pheomelanin in uveal melanoma are suspected to influence the tumoral microenvironment and the antitumoral response [56,57,58,59].

Currently, in cutaneous melanoma, the results of most analyses support a worse prognosis for pigmented lesions [56,57]. Therefore, we decided to see if there were differences in overall survival and disease free survival in two groups of uveal melanomas with different colouration (Kaplan-Meier analysis). We found that both OS (*p* = 0.017) and DSF (*p* = 0.0099) were significantly longer in patients with amelanotic tumours. On the contrary, we did not observe significant differences in overall survival (*p* = 0.21) and disease free survival (*p* = 0.22) between moderately and heavily pigmented tumours. These observations suggested that the finding of any pigment in the tumour area on ophthalmoscopic examination resulted in an increased risk of metastasis and death.

Further analysis was completed to assess whether colouration influences prognosis according to the stage of the disease according to the AJCC. We found that at the lower stage (II, both IIA and IIB), patients with amelanotic tumours live significantly longer (*p* = 0.019), as well as have a longer time to eventual metastasis (*p* = 0.014), than patients with pigmented tumours. In contrast, at higher disease stages (III, both IIIA and IIIB and IIIC) according to the AJCC classification, the degree of colouration of uveal melanoma has no effect on overall survival (*p* = 0.91) and disease free survival (*p* = 0.77). These observations were consistent with the results of McLean et al. in which pigmentation has no effect on prognosis in cases of large and epithelioid cell tumours, i.e., more malignant and advanced [22].

The presence of pigment in uveal melanoma, based on ophthalmoscopic evaluation, was shown as an independent predictor of metastasis and death by Shields et al. Although the authors distinguished three types of pigmentation in a clinical study: pigmented, non-pigmented, and mixed, for detailed analyses (as we did) they adopted two categories: pigmented and non-pigmented uveal melanoma [18]. According to Shields et al., pigmented melanomas have a worse prognosis regardless of tumour size. However, in our study we observed that at higher clinical stages of uveal melanoma pigmentation is no longer relevant. This may be due to unfavourable prognostic changes that occur during tumour mass growth, such as an increased number of epithelioid cells and the accumulation of genetic alterations [5,60,61].

Interesting results have been obtained by assessing the colouration of uveal melanoma and cutaneous melanoma metastases to the liver. These studies found that uveal melanoma metastases showed higher pigmentation scores than cutaneous melanoma metastases, and the level of pigmentation was correlated with different clinical outcomes [62,63,64].

The results of these studies indicate that a higher level of pigmentation in uveal melanoma and its metastasis is related to a higher risk of death.

The results of our analysis should be interpreted bearing in mind some limitations of the method of assessing pigmentation only based on eye fundus examination. In indirect ophthalmoscopy, we only have the possibility of assessing the superficial layers of the intraocular tumour. However, magnetic resonance imaging studies of uveal melanoma and its metastases show that the tumour may have a heterogeneous internal structure in terms of melanin content, which in the clinical picture translates into the presence of varying degrees of pigmentation throughout the tumour volume. The authors of this study claim that most tumours (>80%) are nevertheless homogeneous in terms of melanin content throughout the volume [62,65].

In addition, we did not perform cytogenetic tests to determine the presence of chromosome 3 monosomy, the presence of which is a predisposing factor for metastasis and death. However, in view of our capabilities, we performed an important immunohistochemical assessment of BAP1.

Our study was based on the assessment of tumour pigmentation, which was determined by clinical fundus examination with an indirect ophthalmoscopy, which in daily practice is the easiest to perform and is comfortable for the patient.

Despite this, the results obtained indicating that tumour pigmentation negatively affected prognosis were largely in line with those above, where pigmentation was assessed by microscopic examination. This observation may, in our opinion, have had important implications in clinical practice in qualifying patients diagnosed with uveal melanoma for various therapies, without the need for invasive procedures to obtain material for testing. In addition, numerous studies showed that the degree of pigmentation and associated melanogenesis affect the behaviour of melanoma and the efficacy of local and systemic treatments (chemotherapy, radiotherapy, and immunotherapy) [56,57,66,67,68,69].

In view of the great need to develop an effective therapy for generalized uveal melanoma dissemination and knowing the results of studies indicating a great influence of tumour pigmentation on the planned treatment, further work on this issue is necessary.

## 5. Conclusions

Our results suggest that the prognosis of uveal melanoma depends on the degree of pigmentation that could be assessed by indirect ophthalmoscopy. The presence of pigmentation of the tumours has been associated with the most aggressive form of uveal melanoma and has a more unfavorable prognosis than amelanotic ones. This relationship is significantly evident in less clinically advanced uveal melanomas. These findings could be useful in the choice of therapeutic and follow-up options.

## Figures and Tables

**Table 1:** COMS classification of uveal melanoma pigmentation based on ref. no 21 and 27 [21,27].

	Pigmentation Degree of Uveal Melanoma (COMS)			
	None	Minimal	Moderate	Heavy
Tumours with **uniform** pigmentation	No pigmentation	Cytologic detail evident	Cytologic detail partially obscured	Cytologic detail obscured
Tumours with **variable** pigmentation	No pigmentation	One-third or less pigmented	One-third to two-thirds pigmented	Two-thirds pigmented to complete pigmentation

**Table 2:** Summary statistics for relation between amelanotic and pigmented uveal melanoma and clinical parameters.

Clinical Parameters	Clinical Pigmentation		
	Amelanotic 26 (16.88%)	Pigmented 128 (83.12%)	*p* Value
**Age in years ^a^**	57 (47–62)	61 (54–68)	**0.022**
**Gender ^c^**			0.67
Female	15 (57.69%)	65 (50.78%)	
Male	11 (42.31%)	63 (49.22%)	
**Eye ^c^**			**0.019**
Right	19 (73.08%)	61 (47.66%)	
Left	7 (26.92%)	67 (52.34%)	
**Largest basal tumour diameter ^a^ [mm]**	16.05 (13.72–20.43)	18.20 (15.70–20.20)	0.19
**Greatest tumour height ^a^ [mm]**	10.60 (9.12–11.67)	10.00 (8.07–12.53)	0.53
**Primary tumour (pT) ^c^**			0.10
2	1 (3.85%)	12 (9.38%)	
3	13 (50.00%)	36 (28.12%)	
4	12 (46.15%)	80 (62, 50%)	
**Stage ^c^**			0.092
IIA	1 (3.85%)	9 (7.03%)	
IIB	13 (50.00%)	29 (22.66%)	
IIIA	7 (26.92%)	45 (35.16%)	
IIIB	5 (19.23%)	37 (28.91%)	
IIIC	0 (0.00%)	8 (6.25%)	
**Clinical localization ^c^**			0.42
undefined	0 (0.00%)	1 (0.78%)	
iris and ciliary body	0 (0.00%)	0 (0.00%)	
ciliary body	0 (0.00%)	1 (0.78%)	
ciliary body and choroid	4 (15.38%)	41 (32.03%)	
choroid	21 (80.77%)	81 (63.28%)	
iris, ciliary body, and choroid	1 (3.85%)	4 (3.12%)	
**Shape ^c^**			**0.033**
Dome shape	8 (30.77%)	74 (57.81%)	
Mushroom shape	18 (69.23%)	52 (40.62%)	
Ring	0 (0.00%)	2 (1.56%)	
**Retinal detachment ^c^**			0.11
No RD	2 (7.69%)	29 (22.66%)	
Coexistence of RD	24 (92.31%)	99 (77.34%)	
**Glaucoma ^c^**			0.31
No glaucoma	24 (92.31%)	111 (86.72%)	
Coexistence of glaucoma	1 (3.85%)	17 (13.28%)	
Missing	1 (3.85%)	0 (0.00%)	
**Haemorrhage ^c^**			0.59
No	20 (76.92%)	104 (81.25%)	
Yes	6 (23.08%)	24 (18.75%)	
**Distant metastases ^c^**			0.10
No	22 (84.62%)	86 (67.19%)	
Yes	4 (15.38%)	42 (32.81%)	

^a^*p* value of Wilcoxon two sample test, ^c^ *p* value of Fisher’s exact test. Statistically significant results (*p* < 0.05) are in bold text.

**Table 3:** Summary of statistics for the relation between amelanotic and pigmented uveal melanoma and histopathological parameters.

Histopathological Parameters	Clinical Pigmentation		
	Amelanotic 26 (16.88%)	Pigmented1 28 (83.12%)	*p* Value
**Histologic subtype ^c^**			**0.0013**
Spindle cell melanoma	12 (46.15%)	24 (18.75%)	
Mixed cell melanoma	14 (53.85%)	78 (60.94%)	
Epithelioid cell melanoma	0 (0.00%)	26 (20.31%)	
**Mitotic rate ^a^**			0.39
Median (IQR)	2.50 (1.25–4.00)	3.00 (1.00–6.00)	
Missing	0 (0%)	1 (0.78%)	
**Extraocular growth ^c^**			0.075
No	26 (100.00%)	112 (87.50%)	
Yes	0 (0.00%)	16 (12.50%)	
**Invasion of the optic nerve ^c^**			0.26
No invasion	24 (92.31%)	100 (78.12%)	
Superficial	2 (7.69%)	25 (19.53%)	
Deep with optic nerve	0 (0.00%)	3 (2.34%)	
**Necrosis ^c^**			0.31
No	25 (96.15%)	113 (88.28%)	
Yes	1 (3.85%)	15 (11.72%)	
**Ciliary body invasion ^c^**			0.11
No	21 (80.77%)	81 (63.28%)	
Yes	5 (19.23%)	47 (36.72%)	
**Tumour-infiltrating lymphocytes (TILs) ^c^**			0.87
No	23 (88.46%)	114 (89.06%)	
Yes	3 (11.54%)	14 (10.94%)	
**Scleral infiltration ^c^**			**0.027**
Lack of infiltration	2 (7.69%)	0 (0.00%)	
Intrascleral infiltration	24 (92.31%)	113 (88.28%)	
Extrascleral with ≤5 mm largest diameter	0 (0.00%)	12 (8.59%)	
Extrascleral with >5 mm largest diameter	0 (0.00%)	4 (3.12%)	
**Angioinvasion ^c^**			0.055
Undefined	3 (11.54%)	22 (17.19%)	
No	4 (15.38%)	4 (3.12%)	
Yes	19 (73.08%)	102 (79.69%)	
**Nucleoli size ^c^**			**0.0065**
Lack of nucleoli	2 (7.69%)	4 (3.12%)	
Nucleoli present but inconspicuous	20 (76.92%)	66 (51.56%)	
Prominent nucleoli (macronucleoli)	4 (15.38%)	58 (45.31%)	
**BAP1 status ^c^**			**0.029**
Negative	7 (26.92%)	63 (49.22%)	
Positive	19 (73.08%)	57 (44.53%)	
Missing	0 (0.00%)	8 (6.25%)	

^a^*p* value of Wilcoxon two sample test, ^c^ *p* value of Fisher’s exact test. Statistically significant results (*p* < 0.05) are in bold text.

## Data Availability

The datasets used and/or analyzed during the current study are available from the corresponding author on reasonable request.
